# Does high dose intravenous acetaminophen affect liver function for PDA closure in premature neonate?

**DOI:** 10.1186/s13052-020-00940-2

**Published:** 2021-02-18

**Authors:** Reza Bahrami, Aida Ezzatabadi, Nima Mehdizadegan, Hamid Mohammadi, Hamid Amoozgar, Mohammadreza Edraki

**Affiliations:** 1 Neonatal Research Center, Shiraz University of Medical Sciences, Shiraz, Iran; 2 Department of Pediatrics, Division of Pediatric Cardiology, Nemazee Hospital, Shiraz University of Medical Sciences, Shiraz, 7193711351 Iran; 3 Medical School, Shiraz University of Medical Sciences, Medical School, Shiraz, Iran

**Keywords:** Acetaminophen, Hepatotoxicity, Liver enzymes, Patent ductus arteriosus, Premature infants

## Abstract

**Objectives:**

The aim of this study was to collect consistent data on the efficacy and safety and evaluation hepatotoxicity of intravenous acetaminophen for the treatment of PDA in preterm infants.

**Methods:**

This is an observational longitudinal prospective study on 46 preterm infants with PDA who treated with high dose of acetaminophen and evaluated with echocardiography and serum liver enzymes at Hafez and Zeinabiyeh hospitals from January 2016 to December 2019.

**Result:**

Forty-six preterm infants with PDA treated with intravenous acetaminophen. Rate of closure of PDA was 82.6. There was no significant difference after treatment regarding AST, ALT, Albumin, total and direct bilirubin (*P* value > 0.05) and no adverse side effects were observed in association with intravenous acetaminophen.

**Conclusion:**

High dose of acetaminophen is not more effective than that with standard doses although without hepatotoxic side effect for PDA closure.

## Introduction

A common complication in preterm neonates is patent ductus arteriosus (PDA). PDA is a congenital heart defect that communicate aorta into the pulmonary artery. Patency of PDA is necessary for fetal circulation. In healthy term neonates spontaneous PDA closure happen normally 24–72 h after birth because of increase pressure of oxygen in artery.

Incidence of PDA in preterm neonates between 30 and 37 week gestational age is 10%, those delivered in 25–28 week of GA is 80 and 90% is the percentage of infants born before 24 week GA that after a week would reduce to 2%, 65 and 87% [3, 4].

PDA intervention is controversial, and there is limitation of evidence to guide treatment. There is 3 strategies for closure of PDA in prelatures: Prophylactic management, treatment of clinically detected asymptomatic PDA, and treatment when the PDA is symptomatic neonates. Management of ductal closure include conservative treatments (i.e. fluid restriction, diuretics, etc. and waiting for spontaneous closure), pharmacological management and surgical ligation [1, 4–6].

FDA approved intravenous (IV) indomethacin and ibuprofen (cyclooxygenase inhibitors) as first drug use for treatment of PDA. These drug reduce the levels of prostaglandin that promote ductus arteriosus muscular wall constriction lead to fibrosis as anatomical ductal closure. Prenominated NSAIDs were successful in closure of PDA [7].

NSAID adverse effect include renal function impairment, GI bleeding, necrotizing enterocolitis, intestinal perforation, thrombocytopenia, pulmonary hypertension and hyperbilirubinemia and etc [1, 3].

In recent years increasing acetaminophen administration for PDA treatment because this drug is has same efficacy as NSAIDs with fewer side effects because acetaminophen is prostaglandin synthesis inhibitor with affect at peroxidase site of prostaglandin H synthetase (POX) that differs from COX inhibitor [1, 3].

In neonates who have contraindication for treatment with indomethacin and ibuprofen or the NSAIDs have failed in closure of PDA administration of acetaminophen suggested as a choice before surgical ligation [3].

Evaluating of advantages and disadvantages of pharmacological treatment by assessment of following outcomes: PDA closure failure (according to clinical evaluation or echocardiography criteria) as primary outcome; require surgical ligation of PDA, death, and selected any untoward medical occurrence, as secondary outcomes., not certainly having a causal association with treatment [5].

Prospective trials may support more perception of acetaminophen effectiveness and safety as a further or even as a first-line option for closure of PDA in neonates [8].

Some hepatic side effects have been happened after usage of iv acetaminophen, which may determine a transient raise in liver enzymes or more serious acute liver toxicity [9, 10].

Acetaminophen itself not directly cause of hepatotoxicity in neonates but can be caused by N-acetyl-p-benzoquinoneimine (NAPQI) produced by hepatic cytochrome P450 (CYP) as metabolite production -dependent mixed function oxidase enzyme. The action of NAPQI formation, sulphate elimination, and glucuronide production rate are not known in preterm neonates exactly [11, 12].

The existence of a large therapeutic serum concentration range for acetaminophen suggested by clinical evidence that demonstrate a low or absent hepatic toxicity in neonates [3, 7, 13, 14].

In this study we use high dose acetaminophen in infants with a clinically significant PDA to determine efficacy and hepatic side effects of high dose IV acetaminophen.

## Methods

This is an observational longitudinal prospective study.

The study involved 46 preterm infants (gestational age < 37 weeks, mean birth weight 1099.3 g) with hemodynamically significant patent ductus arteriosus (HsPDA) born at our hospitals (Hafez and Zeinabiyeh) hospitals with the approval of the local ethics committee from January 2016 to December 2019. All patients were admitted in NICU with impression of prematurity.

### Exclusion criteria

Preterm neonates with complex congenital heart disease, those were PDA as life saving for them, the cases who ibuprofen or indomethacin administrated before treatment with acetaminophen, and if the parents did not accept to enroll in this study.

### Treatment eligibility criteria and drug administration protocol

Infants with a gestational age < 37 weeks and who Had clinical signs of significant PDA within the first week of life, diagnosed by pediatric cardiologist were enrolled in the study after obtaining written consent from their parents.

Echocardiography for diagnosis of PDA was done by pediatric cardiologist. We considered PDA as a hemodynamically significant if the patients had at least one of these finding after 3 days or later: Respiratory or cardiovascular compromise (dependency to invasive ventilation or CPAP without RDS or extubation failure), large shunt (LA/AO ratio > 1.5 or sign of LA and LV dilation), PDA larger than 2.5 mm after 1 week, diastolic reversal flow in abdominal aorta after 3 days old (without aortic valve insufficiency), more than 40 mmHg difference between systole and diastole.

Treatment with high dose of intravenous route acetaminophen was started at a dose 20 mg/kg every 6 h for 4 d, with echocardiographic evaluation performed at the end of the treatment. The usual dose of acetaminophen in our center was 10 mg/kg per dose and most references indicates maximum 15 mg/kg/dose q6h for acetaminophen.

Treatment success was defined as complete ductal closure on echocardiography. Pre- and post-treatment levels of liver enzymes (alanine transaminase (ALT), aspartate aminotransferase (AST), albumin, total and direct bilirubin) were measured for evaluation liver toxicity.

Data analysis was done with SPSS version 19 and *p* value < 0.05 consider as significant.

## Result

Forty-six preterm infants were included in this study (January 2016 to December 2019). The median gestational age was 30.1 weeks (minimum–maximum: 25.5–36) and the median birth weight was 1099.3 g (800–3300) (28% below 1000 g and 59% between 1000 to 2000 g) were PDA positive born at our institution enrolled in this study for “first-line” i.v acetaminophen treatment (dose 20 mg/kg every 6 h for 4 d). PDA echocardiographic parameters before starting any i.v acetaminophen treatment are given in Table 1 In this study 23 patients (54%) were intubated and 10 patients was on CPAP. Dependency to ventilator or CPAP were the most common indications for PDA treatment. Only 6 patients had heart failure including clinical sign (poor capillary filling, respiratory distress or gallop rhythm) or echocardiographic features ejection fraction below 50% with dilated LV). Mean ejection fraction in all cases was 61.2 ± 7.6. The most common finding in echocardiography was tricuspid regurgitation (88%). Mitral regurgitation was seen in 23.9% of patients that 2 of them had moderate mitral regurgitation (most probably related to birth time hypoxia). LA/AO ratio was larger than 1.5 in 27 cases.

**Table 1:** Baseline characteristics and echocardiographic data of preterm infants

		Count	Percent	Mean Bitrh weigh(gram)	Gestational age(weeks)
PDA size categories	Small:< 2 mm	14	30.4	1395	30.54
	Medium between 2 to 4 mm	19	41.3	1528.4	30.08
	Large:> 4 mm	9	19.5	1409	29.7
	Total	42	91.3		
Missing System		4	8.7		

Medical treatment of the PDA was failed in 8 patients out of 46 infants and 1 patient because of sepsis, expired during course of treatment. The expired neonate had no sign of liver failure (Hepatomegaly, raising of liver function test or decrease of albumin level. The echocardiography of 46 patients with PDA on treatment cardiac ultrasound resulted in successful closure of PDA among 38 patients (82.6%).

Pre- and post-treatment levels of liver enzymes and bilirubin levels of all infants for the purpose of assessing the treatment’s safety are summarized in Table 2.

**Table 2:** Comparison between value before and after treatment (paired t test)

		Mean Serum level	Number	Std. Deviation	*P*-value
Albumin	Before	3.04	23	0.47	0.672
	After	3.09	23	0.60	
Total bilirubin	Before	5.43	40	3.03	0.258
	After	4.82	40	4.72	
Direct bilirubin	Before	0.46	29	0.16	0.123
	After	0.58	29	0.40	
AST	Before	32.78	31	23.91	0.205
	After	48.55	31	84.26	
ALT	Before	14.85	33	16.21	0.111
	After	19.36	33	18.48	

There was no significant difference after treatment regarding AST, ALT, Albumin, total and direct bilirubin (*P* value > 0.05). Pre- and post-treatment levels of liver enzymes and bilirubin levels were normal in all patients, and no adverse side effects were observed in association with iv acetaminophen. The liver size and clinical examination of the 46 infants during and after treatment were normal. No sign and symptom of hepatotoxicity such as Jaundice, yellowish sclera and hepatomegaly were seen during and after treatment with high dose acetaminophen. Bleeding tendency, GI bleeding and oliguria did not detect. 39 cases cured in first course of acetaminophen administration with clinically improved signs and symptoms of PDA. PDA closure improves dynamic compliance and increases tidal volume in preterm neonates receiving mechanical ventilation and a significant decrease in ventilator setting in our patients with PDA closure than those with failure of PDA closure. Seven infants failed in closure of PDA treated with second course combination acetaminophen and ibuprofen.

## Discussion

Recent results reported on the use of acetaminophen in the treatment of PDA are highly promising, but adequately powered. The aim of this study is to collect consistent data on the efficacy and safety of high dose intravenous acetaminophen for the treatment of PDA in preterm infants. Drugs like cyclooxygenase (COX) inhibitors, e.g., indomethacin and ibuprofen, were used for closure of PDA. Acetaminophen is an alternative therapeutic approach for ductal closure through inhibition of prostaglandin synthetase activity. Although its efficacy in PDA closure has been approved [7]. Acetaminophen seems to inhibit peroxidase segment of the enzyme prostaglandin synthetase, unlike NSAIDs that inhibit cyclooxygenase pathway of this enzyme. NSAIDs are associated with significant adverse effects, including peripheral vasoconstriction, gastrointestinal bleeding and perforation, renal failure, oliguria and impaired platelet aggregation or inhibition of bilirubin glucuronidation in the liver and hyperbilirubinemia. These adverse effects emphasize the possible benefits of alternative treatment with acetaminophen for PDA management [8].

We used acetaminophen as a first line in the treatment of PDA for 46 patients successfully without any significant complication. Our study showed that acetaminophen is effective in promoting ductal closure of PDA in preterm infants with82.6% closure rate that was comparable with other treatment options in other studies. El mashad et al. 2017 showed The rate of closure in acetaminophen therapy in 100 neonates (80%) was more or less similar to that after ibuprofen (77%) and indomethacin (81%) therapy [7]. Hammerman et al. reported that they used acetaminophen in five cases because of different contraindications and unresponsiveness to the treatment. At all cases, the ducts were closed and positive responses were observed to the acetaminophen treatment [15]. Oncel et al. have reported eight cases who were unresponsive to ibuprofen or ibuprofen was contraindicated and received acetaminophen with successful PDA closure [16].

Even higher rate of PDA closure (> 95%) was reported by some other investigators. In the study of Dash et al. enteral acetaminophen showed a PDA closure rate of 100% and no hepatotoxicity was detected. This surprising high result about acetaminophen efficacy deviates from other studies’ results, but it must be considered that this RCT evaluated patients showing a mean GA of 31.6 weeks, higher than neonates in other trials. With better response to pharmacological treatment [17] PDA is known to be less responsive to cyclooxygenase inhibition in young preterm neonates due to higher expression of prostaglandin receptors in their PDA walls. H¨arkin et al. demonstrated a faster PDA closure rate in acetaminophen group (95%) than in placebo group. The authors used a different drug dosage, administering 20 mg/kg of acetaminophen at 24 h of life, followed by 7,5 mg/kg every 6 h for 4 days and the ductus closed at a mean of 177 h of postnatal life in treated patients versus 338 h in controls. However, GA influenced ductal closure; in fact, in extremely preterm infants (< 27 weeks’ GA), acetaminophen did not show a significant effect; among these, 4 preterms (50%) required PDA ligation [18]. Le et al. [1] agree with the idea that acetaminophen seems to be a good alternative in PDA treatment and should be considered, in case of ibuprofen contraindication, before ligation. The author also recommends performing other trials because two studies published on 2013 found low iv acetaminophen success rate in small groups of patients (*n* = 29 and *n* = 3Roofthooft et al. [19] had disappointing results with PDA closure after iv acetaminophen treatment with a low success rate of only 17%. This could be due to a late start of acetaminophen administration in their study (median of 14 days). But El Kuffash et al. [20] evaluated late treatment with iv acetaminophen beyond the 2nd week of life which became effective in PDA closure, avoiding PDA ligation.

In this study we try to use higher dose of acetaminophen and although we have no further complication related to this dose it did not lead to higher rate or PDA closure in compare to similar studies with lesser dose..The liver size and clinical examination of the 46 infants during and after treatment were normal.This result was in agreement with Jacqz-Aigrain et al. [14] who reported that neonates tend to suffer less from the hepatotoxic effects of acetaminophen than do older children. Hammerman et al. reported that acetaminophen could offer important therapeutic advantages over NSAID (e.g., indomethacin and ibuprofen) as acetaminophen has no peripheral vasoconstrictive effect, so it can be given to infants with clinical contraindications to NSAIDs [5]; But it seems higher dose of acetaminophen dose not promote this safe benefit and the optimum dose to achieve this effect is 10 to 15 mg per kg weight.

In our study bleeding tendency and GI complication did not detect. GI bleeding never seen that went with the results of other investigators. A safer profile in terms of gastrointestinal bleeding and hyperbilirubinemia after acetaminophen administration instead of ibuprofen has been described by Evans [21] and Terrin et al. [22] In contrast to our results. Dash et al. [17] reported striking high intestinal bleeding rate in the acetaminophen group (26.3%) The high intestinal bleeding rate in their study may be related to high osmolality of acetaminophen used in their study.

### Limitation of this study

Due to significant cost and need of multiple sampling at specific time to achieve a reliable blood level of acetaminophen we did not perform acetaminophen blood level measurement for our patients. This study focus on short term hepatotoxicity effect of acetaminophen but it is worth to mentioned that acetaminophen has neurocognitive and fertility effect that need long term follow up and is beyond the scope of this article but it should be evaluated in a long term study. Also, the extend of hepatotoxicity could be evaluate by some less familiar testes such as acylcarnitines that were not performed in this study mostly due to need of more sampling in premature cases.

## Conclusion

The goal of the studies on PDA management would be to perform an individualized therapy, choosing the for each of the patient characteristics, which could be the most effective as much as possible, personalized, and with the lowest side effects. Acetaminophen is as effective as indomethacin and ibuprofen in closure of PDA in preterm neonates with less side effects than both in compare to result of other studies. But higher dose than 10 to 15 mg/kg/dose did not lead to higher rate of PDA closure and not recommended in addition of safe side effect profile with 20 mg/kg/dose and also it is safe in case of liver function based on liver function test in this study.

## Data Availability

We state that the data used and/or analyzed during the current study are available from the corresponding author on reasonable request. Data sharing is applicable to this article and datasets were generated and analyzed during the current study and data sharing is allowed.

## Abbreviations

PDA: Patent ductus arteriosus
LFT: Liver function test
LA: Left atrium
LV: Left ventricle
I.V: Intravenous
COX inhibitors: Cyclooxygenase inhibitors
